# Histamine H4 receptor gene polymorphisms: a potential contributor to Meniere disease

**DOI:** 10.1186/s12920-019-0533-4

**Published:** 2019-05-27

**Authors:** Danxia Qin, Han Zhang, Jiehua Wang, Zhuquan Hong

**Affiliations:** 1 Inpatient Department 7th floor District 6, Dongjie Branch of Quanzhou 1st Hospital, Quanzhou 1st Hospital Affiliated to Fujian Medical University, Quanzhou, 362000 Fujian China; 2 Inpatient Department 10th floor District 13, Chendong Branch of Quanzhou 1st Hospital, Quanzhou 1st Hospital Affiliated to Fujian Medical University, Quanzhou, 362000 Fujian China

**Keywords:** Meniere’s disease, Histamine H4 receptors, Polymorphism, Proinflammatory cytokines

## Abstract

**Background:**

The immune system is likely involved in the pathophysiology of Meniere’s disease (MD). However, its role of patients with MD has not been well studied. Given that histamine H4 receptors are highly expressed in immune system, we tested the hypothesis that histamine H4 receptor gene polymorphisms are a potential contributor to the risk of MD.

**Methods:**

A group of patients was enrolled with a diagnosis of definite MD based on the American Academy of Otolaryngology-Head and Neck Surgery Committee on Hearing and Equilibrium guidelines and a control group of patients without any vestibular disease. We selected one SNP, rs77485247 in *HRH4* and conducted an exploratory investigation of its correlations with the symptoms of vertigo and proinflammatory cytokines levels in MD patients.

**Results:**

*HRH4* rs77485247 polymorphism may be associated with the risk of MD. Furthermore, basal levels of proinflammatory cytokines, such as IL-1β and TNF-α, in PBMCs are increased in patients with MD compared to control patients. This increased basal level of proinflammatory cytokines is prominent in MD patients with the A allele.

**Conclusions:**

These suggested that *HRH4* rs77485247 polymorphism may be an important mediator in regulating proinflammatory cytokines, which are involved in the pathogenesis of MD.

## Background

Meniere’s disease (MD) is a peripheral vestibular disorder and it is characterized by fluctuating hearing loss, tinnitus, and vertigo [1]. While the pathological mechanism underlying MD is still poorly understood, many studies have postulated that various factors may be involved, including endolymphatic hydrops, allergy, inflammation, infection, and autoimmune disorders [1, 2]. Among these theories, it seems that the immune system plays a critical role in a large population of MD patients [3, 4]. However, the role of the immune system in patients with MD has not been well studied.

Histamine receptors are critically involved in the function of human immune system, and the pathophysiology of MD, which is evident by the effectiveness of antihistamine treatment (e.g. Betahistine) in MD patients [5]. Betahistine is an approved treatment for MD and it associated vertigo symptoms. Betahistine is a weak agonist for histamine H1 receptors and an antagonist for H3 receptors [6]. Previous animal studies have shown that Betahistine can improve vestibular compensation in animal models by promoting blood flow of vestibulocochlear and decreasing excitatory responses induced by histamine in vestibular cells [6]. In human MD patients, multiple studies have shown that betahistine can reduce the frequency and severity of vertigo, and improve symptoms of vertigo, such as vomiting and nausea [7]. Interestingly, recent animal studies also pointed out that histamine H4 receptor antagonists have a pronounced inhibitory effect on mammalian vestibular neuron activity [8], indicating an important pharmacological target for MD. However, no study so far has been conducted to investigate the involvement of histamine H4 receptors in human MD. Importantly, many studies have demonstrated that histamine H4 receptors play a key role in immune and inflammatory disorders [9]. Given that histamine H4 receptors are highly expressed on peripheral blood mononuclear cells (PBMCs) [9], and increasing evidence showed that proinflammatory cytokines contribute to pathogenesis of MD [10], we hypothesized that histamine H4 receptor gene polymorphisms is a potential contributor to MD.

To test this hypothesis, a group of patients was enrolled with a diagnosis of definite MD based on the American Academy of Otolaryngology-Head and Neck Surgery (AAO-HNS) Committee on Hearing and Equilibrium guidelines [11] and a control group of patients without any vestibular disease. Based on previous reported that *HRH4* polymorphisms are involved in allergy, inflammation, and immune reaction [12], one SNP rs77485247 was selected, which has been shown to be associated with the risk of allergic rhinitis [13], a nasal allergy condition that is associated with high prevalence of inner-ear symptoms, including MD [14]. We conducted an exploratory investigation of the correlations of rs77485247 with the symptoms of vertigo and proinflammatory cytokines levels in MD patients.

## Methods

### Human subjects

Ninety MD patients were enrolled at Quanzhou 1st Hospital Affiliated to Fujian Medical University from January 2016 to December 2017. These patients all had definite MD according to the American Academy of Otolaryngology-Head and Neck Surgery Committee on Hearing and Equilibrium guidelines [11]. A second group of 90 patients who did not exhibit any vestibular disease (control group) was also enrolled. This control group of patients had other otolaryngologic reasons to attend our facilities during the same time of this project as MD patients. The clinical data of MD patients were inspected by on-site physicians to determine the disease severity. We recorded the number of vertigo attacks, the frequency (per year) of the vertigo attacks, and the duration (months) of symptoms as indicators of severity of vertigo. In addition, the worst hearing level at attack periods for patients was expressed by the four frequencies 0.5, 1, 2, and 4 kHz averages in pure-tone audiometry and was divided into four stages according to AAO-HNS. For all patients, demographic factors, mental illnesses and medical comorbidity were collected (Table 1). The data collection and analysis of demographic factors, comorbidity and mental disorders were conducted by separate researchers who were blinded to the following study procedures. We excluded patients who have missing medical notes. All patients signed the informed consent, which was approved by the ethical committee of Quanzhou 1st Hospital Affiliated to Fujian Medical University.

**Table 1:** Characteristics of human subjects

Variables	Control (*n* = 90)	Bilateral or Unilateral MD (*n* = 90)	*P*-value
Age, Mean (SD)	60 (12.3)	62 (12.8)	0.12
Gender, n (%women)	57 (63.0)	48 (52.5)	0.34
Ischemic heart disease, n (%)	6 (6.7)	9 (10.0)	0.68
Smoking, n (%)	9 (10.0)	7 (7.8)	0.68
Chronic obstructive pulmonary disease, n (%)	12 (13.3)	15 (16.7)	0.81
Rheumatoid history, n (%)	6 (6.7)	27 (30.0)	0.02*
Hypertension, n (%)	30 (33.3)	42 (40.0)	0.34
High cholesterol, n (%)	27 (30.0)	30 (33.3)	0.47
Diabetes, n (%)	15 (16.7)	39 (43.3)	0.01*
Asthma, n (%)	6 (6.7)	21 (23.3)	0.03*
Osteoarthritis, n (%)	6 (6.7)	48 (53.3)	0.01*
Thyroid disease, n (%)	3 (3.3)	3 (3.3)	1.00
Irritable bowel syndrome, n (%)	9 (10.0)	3 (3.3)	0.11
Hiatus hernia, n (%)	3 (3.3)	6 (6.7)	0.38
Gastroesophageal reflux disease, n (%)	21 (23.3)	24 (26.7)	0.55
Anxiety, n (%)	6 (6.7)	33 (36.7)	0.02*
Depression, n (%)	9 (10.0)	27 (30.0)	0.03*

Age was compared by unpaired Student’s t test. Qualitative variables were compared by Chi-squared test. Asterisks represent statistical significance

### Extraction of DNA from blood samples

Fasting peripheral venous blood samples (4 mL) were collected from the patients. To isolate peripheral blood mononuclear cells (PBMC), 2 ml blood were used at our on-site clinical research center (see below for details). Additional 2 mL blood was used to extract DNA using genomic DNA extraction kits (CAS# B518205–0025, Sangon Biotech, Shanghai, China) based on the instructions of the manufacturer. An ultraviolet spectrophotometer was then used to assess the purification, concentration and recovery of small fragments of DNA. To be satisfied for further analysis, the OD260/OD280 ratio was set to be 1.7–1.9. The DNA extracts were stored at − 20 °C until further analysis.

### PBMC isolation and cytokine measurement

PBMCs were isolated using an established method as previously described. PBMCs were cultured in RPMI 1640 supplemented with 10% (v/v) fetal bovine serum (Sigma-Aldrich, St. Louis, MO). Cells were seeded in 12-well plates at 1 × 10^6^ cells/mL, followed by incubation for 16 h in 7% CO_2_ at 37 °C. PBMCs were then centrifuged, and supernatants were collected and stored at − 20 °C. Frozen samples were allowed to be thawed immediately prior to analysis. Human Cytokine Magnetic 35-Plex Panel (CAS# LHC6005M, ThermoFisher Scientific, Waltham, MA USA) that is commercially available was used to quantify the levels of IL-1β and TNFα in conditioned supernatant based on the protocols of the manufacturer, which were provided by Invitrogen™. A Luminex detection system (MAGPIX 200) with xPONENT™ software 3.1 was used for data recording.

### Genotyping

We designed the primers of rs77485247 in *HRH4* and Sangon Biotech Co., Ltd. (Shanghai, China) synthesized the primers, the sequence of which was shown in Table 2. 0.1 μg genomic DNA was used for polymerase chain reaction (PCR). The conditions for amplification were optimized. Pre-denaturation was at 94 °C for 3 min, followed by 40 cycles. Each cycle included a denaturation at 95 °C for 35 s, an annealing at 60 °C for 35 s and at 72 °C for 30 s. After 40 cycles, an extension was at 72 °C for 10 min. A DNA gel analysis system (TLN Technology Co., Ltd., Beijing, China) was used to analyze the purification of the final products. To sequence the final PCR products, purified products were sent to Invitrogen (Shanghai, China). After obtaining the results of the product sequence, we compared the results with NT-010966.13 sequence available at the National Center for Biotechnology Information (NCBI) using Basic Local Alignment Search Tool (BLAST; https://blast.ncbi.nlm.nih.gov/Blast.cgi) to further determine individual genotype.

**Table 2:** Primer sequences of the amplified fragments

SNP	Sequence (5′ to 3′)	Product size (bp)
rs77485247 T *>* A (ss142022671)	F: TGGAGATGGATGCATCACAG R: TCCCCAGTTGTATACAGTC	566

### Statistical methods

SPSS 21.0 statistical package (SPSS Inc., Chicago, IL, USA) was used for data analysis. Data were expressed as the mean ± standard deviation, and a t test or one-way ANOVA was used to compare between the groups of normal distribution, as appropriate. Enumeration data were shown in percentage, and chi-square test or Fisher’s exact test was used to test Hardy-Weinberg equilibrium. The odds ratio (OR) and 95% confidence interval (CI) were used to describe the relative risk of genotypes and alleles. Multivariable logistic regression analyses were performed for the adjusted associations between coffee type consumed and hearing loss or tinnitus. A value of α less than 0.05 was significant.

## Results

### Comparisons of baseline characteristics between the control group and MD group

Demographic factors, comorbidity and mental disorders were shown in Table 1. We did not find any difference in age or gender between MD patients and the control patients. At the time of diagnosis of MD, comorbidities included ischemic heart diseases, chronic obstructive pulmonary disease, rheumatoid history, hypertension, high cholesterol, asthma diabetes mellitus, thyroid disease, osteoarthritis, hiatus hernia, irritable bowel syndrome, and gastroesophageal reflux disease. We also included depression and anxiety. The prevalence of rheumatoid history, asthma, diabetes mellitus, and osteoarthritis was significantly higher (*p* < 0.05) in MD groups as compared with control group. In addition, the prevalence of mental disorders (anxiety and depression) in MD patients was also significantly higher than those in control group (*p* < 0.05).

### Distribution of genotype and allele frequencies of rs77485247 in *HRH4* gene

We tested Hardy-Weinberg balance in MD patients and control patients and found that the distribution of genotype and allele frequencies was consistent with Hardy-Weinberg balance. These results indicated that the enrolled groups of patients were representative. As shown in Table 3, there are also significant differences between the distribution frequencies of mutant genotype (TA + AA) and those of A allele of rs77485247 in MD patients and control patients.

**Table 3:** Comparison of the genotype and allele distributions of *HRH4* rs77485247

SNP	MD group (*n* = 90) (n, [%])	Control group (*n* = 90) (n, [%])	*χ*2	*p*	OR (95% CI)	Adjusted p*	Adjusted OR* (95% CI)
rs77485247 T *>* A							
TT	68 (75.6)	80 (88.9)			1		1
TA	19 (21.5)	9 (10.0)	8.529	0.033	2.58 (1.34–4.97)	0.027	2.06 (1.28–4.42)
AA	3 (3.3)	1 (1.1)	2.760	0.182	5.34 (0.58–48.54)	0.031	3.45 (0.78–9.46)
TA + AA	22 (24.4)	10 (11.1)	10.380	0.012	2.75 (1.46–5.17)	0.022	2.55 (1.39–4.76)
T	155 (86.7)	169 (94.4)			1		1
A	25 (13.3)	11 (5.6)	11.570	0.011	2.67 (1.49–4.78)	0.014	2.58 (1.74–5.03)

*CI* confidence interval, *HRH4* histamine H4 receptor, *OR* odds ratio, *SNP* single nucleotide polymorphism. * Adjusted for rheumatoid history, diabetes, asthma, osteoarthritis, anxiety, and depression

### The associations of rs77485247 in *HRH4* gene with vertigo symptoms of MD patients

We evaluated patient clinical data and rs77485247 genotypes among the MD patients. MD patients were divided into two groups; one group of patients with the A allele (mutant homozygote (AA + TA) and the other group of patients with wild-type homozygote TT genotype. The averages of the number or the frequency of vertigo attacks and duration of vertigo symptom are shown in Table 4. There was significant difference in the number or the frequency of vertigo attacks and duration of vertigo symptom between TA + AA group and TT group.

**Table 4:** Relation between genotype of *HRH4* rs77485247 and vertigo

Variables	Genotype	n	Frequency of vertigo attacks (times/year) (SD)	Mean hearing level (SD)	Duration of symptom (months) (SD)
rs77485247 T *>* A	TT	68	1.6 (1.3)	38.4 (3.7)	37.6 (3.8)
	TA + AA	22	2.7 (1.2)*	40.2 (4.9)	46.5 (4.2)*

Asterisks represent statistical significance compared to TT genotype (*p* < 0.05)

### The associations of rs77485247 in *HRH4* gene with cytokine levels in PBMCs of MD patients

We also evaluated patient cytokine levels in PBMCs. MD patients were divided into two groups; one group of patients who have A allele (mutant homozygote AA + TA and the other group of patients who have wild-type homozygote TT genotype. The averages of the basal levels of IL-1β (pg/mL) and TNF-α (pg/mL) in PBMCs are shown in Table 5. There was significant difference in the basal levels of IL-1β and basal levels of TNF-α between TA + AA group and TT group, and the basal levels of IL-1β and TNF-α were higher in MD.

**Table 5:** The associations between *HRH4* rs77485247 and biochemical parameters of MD patients

Variables	Genotype	n	Basal Levels of IL-1β (SD), pg/mL	Basal Levels of TNF-α (SD), pg/mL
rs77485247 T *>* A	TT	68	2.5 (3.3)#	5.9 (7.4)#
	TA + AA	22	6.4 (7.2)*#	13.8 (9.8)*#
Control		90	0.99 (2.1)	3.1 (6.5)

Asterisks represent statistical significance compared to TT genotype (*p* < 0.05). Ponds represent statistical significance compared to control (*p* < 0.05)

## Discussion

Our study found that *HRH4* rs77485247 polymorphism may be associated with the risk of MD. Furthermore, basal levels of proinflammatory cytokines, such as IL-1β and TNF-α, in PBMCs are increased in patients with MD compared to control patients. This increased basal level of proinflammatory cytokines is prominent in MD patients with the A allele. These results suggested that *HRH4* rs77485247 polymorphism may be an important mediator in regulating proinflammatory cytokines, which are involved in the pathogenesis of MD.

*HRH4* is located in chromosome 18 ql1.2 and it contains 2 introns (> 7 kbp) and 3 exons with its corresponding mRNA of 3.689 kb (NM 021624) [15]. It has been demonstrated that *HRH4* mRNA expresses widely in various peripheral tissues [16]. Notably, *HRH4* is preferentially expressed in inflammatory-response-related tissues, including peripheral blood cells, bone marrow, spleen, small intestine, and lung [17]. Notably, the prevalence of rheumatoid history, asthma, diabetes mellitus, and osteoarthritis in our enrolled MD groups as compared with control group. These results are consistent with previous reports that these disorders are common comorbidities of MD [18]. Hence, *HRH4* polymorphisms may be a linker between inflammatory disorders and pathophysiology of MD. Indeed, a few studies have reported that *HRH4* polymorphisms are involved in multiple disorders, such as allergic rhinitis [13], ankylosing spondylitis [19], atopic dermatitis [20], asthma [21]. These studies reported the involvement of multiple *HRH4* polymorphisms in these disorders. In the present study, while we focused on *HRH4* rs77485247 polymorphism, future studies will be necessary to conduct a more comprehensive investigation on multiple *HRH4* polymorphisms and understand not only the association of these polymorphisms with the risk of MD, but their associations with and the efficacy of antihistamine treatment of MD patients.

While our study focused on IL-1β, it should be noted that three cytokines are encoded by the human IL-1 complex genes, not only including IL-1β, but also IL-1α and IL-1RA, the antagonist receptor. It is known that the balance between the levels of IL-1 RA and IL-1β reflects the severity of inflammation. Consistent with previous study [22], we found that IL-1β levels are very low in PBMCs of healthy individuals. Remarkably, we also found that IL-1β basal levels were elevated in MD patients, more prominently in MD patients who have an allele of *HRH4* rs77485247. This phenomenon was similarly observed in TNF-α basal level in PBMCs. Although the evidence to validate our findings is limited in MD patients, it has been shown in a case control study that 21% of patients with MD had elevated levels of pro-inflammatory cytokines such as IL-1β, IL-1RA, TNFα, and IL-6 in the supernatant from patient-derived peripheral blood mononuclear cells [23]. While it is not clear the consequence of elevated basal levels of TNF-α and IL-1β in PBMCs of MD patients, we postulated that this might be mediated by H4 receptor induced cytokine production. Accumulating evidence shows that H4 receptor in immune system plays an immunomodulatory role in the production of cytokines [24–26]. Specifically, H4 receptor is involved in pathophysiological mechanisms of various inflammatory conditions such as asthma, autoimmune diseases, and other allergic disorders. Hence, it is likely that *HRH4* rs77485247 polymorphism may be involved in the maintenance of inflammation by regulating inflammatory signals [27–30].

Adding to the literature, our study provided additional evidence that MD represents a heterogeneous disorder, which is likely associated with altered basal levels of proinflammatory cytokines in immune system that are mediated by H4 receptor. These results may explain why betahistine treatment has only good response in some MD patients, but not in others [31], since the antihistaminic effects of betahistine are thought to be mediated by being a weak H1 receptor agonist and a potent H3 receptor antagonist. Interestingly, studies on human endolymphatic sac tissue (ES) found that the expression of the histamine receptor gene *HRH1* was primarily in the epithelial lining of the ES, whereas *HRH3* was expressed exclusively in the sub-epithelial capillary network. *HRH2* or *HRH4* were not expressed [6]. These results together indicated that betahistine effects on MD may primarily be mediated by the antagonism of H3-receptor, resulting in the inhibition of vestibular neurotransmission. When considering the potential contributions of H4-receptor mediated immune-regulation, as indicated by our study, we may postulate that diverse populations of histamine receptors are likely involved in the pathophysiology of MD. Therefore, future antihistaminic therapeutics for the treatment of MD should consider these subpopulations of histamine receptors.

The main strengths of our study include perspective enrollment of patients and direct comparison of patients with definite MD with any control patients who had no vestibular disease. However, there are several limitations of our study. First, a major limitation of the present study is that we only enrolled a limited number of patients for each group (*N* = 90 per group) in an adjacent area. Like many available case-control studies on MD, a relatively small number of patients were reported. This is likely due to the epidemiological issue because of the challenges of enrolling patients with MD for data collection and analysis. To the best of our knowledge, there are only a few studies of MD available with larger cohorts [10, 18, 32, 33]. Another limitation is that the population of patient in our study was primarily eastern Asian Chinese and cannot represent the other populations that may have higher risk of MD, such as Caucasian population. For example, in a study that was conducted in the US, Caucasians have a higher prevalence than other major ethnicities, including Hispanics, Blacks, and Asians [34]. Given that histamine H1 receptor expression exhibits genetic differences relative to race [35], we cannot rule out the possibility that there are racial predispositions to *HRH4* rs77485247 polymorphism in MD patients. Hence, further study will be needed to validate our findings in a larger and more diverse population of patients.

## Conclusion

In summary, our exploratory study reported an interesting finding that *HRH4* rs77485247 polymorphism may be associated with the risk of MD. Together with recent animal study that demonstrated the efficacy of selective H4 receptor antagonisms in mammalian vestibular neuron activity [8], our results suggested that heterogenous populations of H4 receptor may be involved in the pathophysiology of MD. Furthermore, we reported that MD patients exhibited increased basal levels of TNF-α and IL-1β in PBMCs. Particularly, individual patient with MD who had prominent elevated basal levels of I TNF-α and IL-1β, may benefit from target therapies to block TNF-α or IL-1β. Taken together, our study supported that MD is a heterogenous disorder and future research on therapeutic development should consider multimodal approach, which is likely to have improved treatment outcome than currently available monotherapy (e.g., betahistine).

## Abbreviations

MD: Meniere’s disease
PBMCs: Peripheral blood mononuclear cells
